# Friends or Foes: Matrix Metalloproteinases and Their Multifaceted Roles in Neurodegenerative Diseases

**DOI:** 10.1155/2015/620581

**Published:** 2015-10-11

**Authors:** Marjana Brkic, Sriram Balusu, Claude Libert, Roosmarijn E. Vandenbroucke

**Affiliations:** ^1^Inflammation Research Center, VIB, 9052 Ghent, Belgium; ^2^Department of Biomedical Molecular Biology, Ghent University, 9052 Ghent, Belgium; ^3^Department of Neurobiology, Institute for Biological Research, University of Belgrade, 11060 Belgrade, Serbia

## Abstract

Neurodegeneration is a chronic progressive loss of neuronal cells leading to deterioration of central nervous system (CNS) functionality. It has been shown that neuroinflammation precedes neurodegeneration in various neurodegenerative diseases. Matrix metalloproteinases (MMPs), a protein family of zinc-containing endopeptidases, are essential in (neuro)inflammation and might be involved in neurodegeneration. Although MMPs are indispensable for physiological development and functioning of the organism, they are often referred to as double-edged swords due to their ability to also inflict substantial damage in various pathological conditions. MMP activity is strictly controlled, and its dysregulation leads to a variety of pathologies. Investigation of their potential use as therapeutic targets requires a better understanding of their contributions to the development of neurodegenerative diseases. Here, we review MMPs and their roles in neurodegenerative diseases: Alzheimer's disease (AD), Parkinson's disease (PD), amyotrophic lateral sclerosis (ALS), Huntington's disease (HD), and multiple sclerosis (MS). We also discuss MMP inhibition as a possible therapeutic strategy to treat neurodegenerative diseases.

## 1. Introduction 

In the past two decades, the function of matrix metalloproteinases (MMPs) in the central nervous system (CNS) has gained much attention. MMPs are calcium (Ca^2+^) dependent zinc (Zn^2+^) containing endopeptidases produced in latent forms. Once activated, they participate in the regulation of diverse physiological and pathological processes [1]. MMPs are involved in the degradation of extracellular matrix (ECM) components, remodeling of tissues, shedding of cell surface receptors, and processing of various signaling molecules. MMPs are essential for brain development due to their association with important neurophysiological functions, such as synaptic plasticity [2, 3] and long-term potentiation [4, 5]. In the adult brain under normal conditions, MMPs are expressed at basal but detectable levels. Increased expression of MMPs is observed in a variety of pathological conditions, including neurodegenerative diseases such as Alzheimer's disease (AD), Parkinson's disease (PD), amyotrophic lateral sclerosis (ALS), Huntington's disease (HD), and multiple sclerosis (MS) and in neuroinflammatory conditions such as traumatic brain injury, stroke, and meningitis. As part of the neuroinflammatory response, MMP activity at CNS barriers contributes to the increase in permeability by altering the properties of ECM and tight junctions. This results in aggravation of neuroinflammation-induced brain damage. On the other hand, activation of MMPs is known to help in tissue repair, angiogenesis, and neurogenesis. In this review, we focus on both beneficial and detrimental roles of MMPs in neurodegenerative diseases.

## 2. Matrix Metalloproteinases (MMPs)

**Figure 1:**
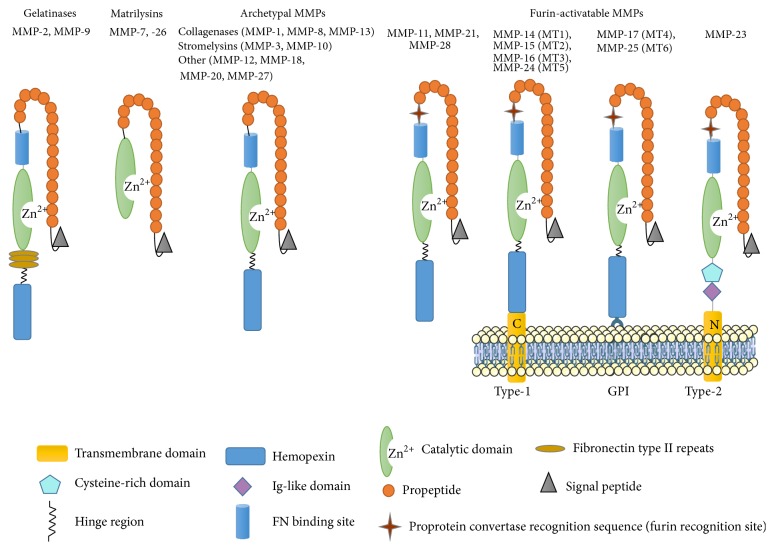
Classification of the MMPs based on their domain organization. MMPs are grouped into four major groups: gelatinases, matrilysins, archetypal MMPs, and furin-activatable MMPs. The typical structure of MMPs consists of a signal peptide, propeptide, a catalytic domain, hinge region, and a hemopexin domain. In addition, members of the gelatinases family have extra fibronectin type II motif repeats in the catalytic domain, and matrilysins have neither a hinge region nor hemopexin domains. Furin-activatable MMPs contain a furin recognition motif and are subcategorized into either secreted or membrane bound. Based on the type of membrane attachment, they are subdivided into type I transmembrane MMPs, GPI-linked MMPs, and type II transmembrane MMPs. Type-II transmembrane MMPs lack a cysteine switch. Instead, they have a cysteine rich domain and IgG-like domain. C, C-terminal domain; FN, fibronectin; GPI, glycophosphatidylinositol; MMP, matrix metalloproteinases; N, N-terminal domain.

MMPs, together with adamlysins and astacins, belong to metzincins, a family of Zn^2+^-dependent, Ca^2+^-containing endopeptidases (24 members in mammals) [1]. MMPs are multidomain proteins consisting mostly of the following domains: N-terminal signal peptide (which is cleaved in the secretory pathway), propeptide (which maintains latency of MMPs), catalytic domain (holds the Zn^2+^ ion), hinge region (connecting sequences), and C-terminal hemopexin-like domain (required for substrate and TIMP recognition) [6] (Figure 1). Besides these common domains, some MMPs have alternative peptide structures and different additional domains [7]. MMPs are produced as zymogens (pro-MMP) that are activated by other enzymes or free radicals through the cysteine switch mechanism. The thiol group of a cysteine residue in the N-terminal prodomain binds to and blocks the active-site Zn^2+^ atom; activation occurs when the thiol group is blocked or removed [8]. It has been shown that MMPs play an important role in various physiological and pathological processes in the body. Active MMPs can degrade ECM components. ECM is a dynamic structure that supports multiple physiological processes. It acts as an adhesion site for various cells and serves as a storage site for different signaling molecules, growth factors, and proteins in general, thus influencing development and migration of the cells. ECM consists mainly of glycosaminoglycans, proteoglycans, and fibrous proteins (collagen, laminin, and fibronectin). MMP cleavage of ECM influences cell migration, embryogenesis, and other processes during development as well as in the adult organism. In the brain, MMPs are involved in tissue remodeling after injury, neurogenesis, axonal growth, angiogenesis, CNS barrier disruption, myelinogenesis, and demyelination. Additionally, MMPs play an active role in immune processes by cleaving various molecules, including growth factors, death receptors, chemokines, and cytokines [9, 10]. For example, several MMPs can activate tumor necrosis factor (TNF) [11–15] and transforming growth factor-beta (TGF-*β*) [16], while other MMPs degrade interleukin-1*β* (IL-1*β*) [17]. Ultimately, MMP cleavage of chemokines and cytokines can lead to either pro- or anti-inflammatory processes [18].

Based on their domain organization, MMPs are classified into four major subgroups: (1) gelatinases (MMP-2, MMP-9), (2) matrilysins (MMP-7, MMP-26), (3) archetypal MMPs, and (4) furin-activated MMPs (Figure 1 and Table 1). The archetypal MMPs contain different types: stromelysins (MMP-3, MMP-10), collagenases (MMP-1, MMP-8, MMP-13), and other MMPs (MMP-12, MMP-18, MMP-20, MMP-27). Similarly, the furin-activated MMPs are divided in secreted MMPs (MMP-11, MMP-21, MMP-28); type-I transmembrane MMPs (MMP-14, MT1-MMP, MMP-15, MT2-MMP, MMP-16 (MT3-MMP), and MMP-24 (MT5-MMP)), type-II transmembrane MMPs (MMP-23), and GPI-anchored MMPs (MMP-17 (MT4-MMP), MMP-25 (MT6-MMP)).

**Figure 2:**
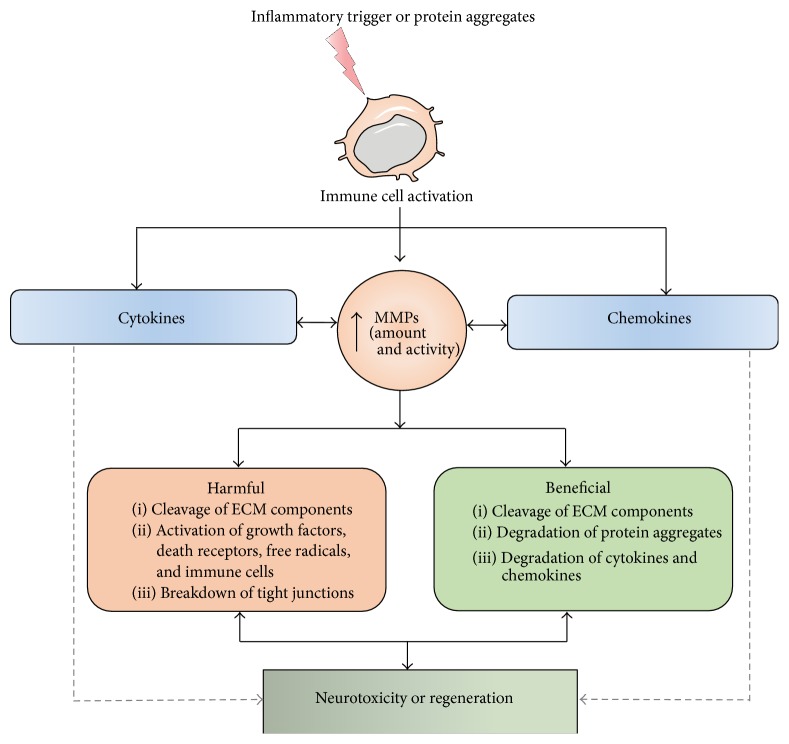
Schematic representation of the activation of MMPs, their interactions with cytokines and chemokines, and the outcome of the interactions. MMPs are induced and activated in the presence of an external trigger (e.g., inflammatory stimuli) or abnormal proteins (e.g., protein or peptide aggregates). The activated MMPs can alter the properties of cytokines and chemokines. They also interact with the extracellular matrix, cell surface receptors, growth factors, integrin, signaling molecules, and tight junction proteins and alter their properties. This affects neuroinflammation, cell death or survival, growth, and regeneration. ECM, extracellular matrix; MMP, matrix metalloproteinase; TJs, tight junctions.

MMPs are stringently regulated because in their active state they can be detrimental. MMPs are modulated on several levels: transcriptional activation, removal of the prodomain, interaction with ECM components, and inhibition by endogenous inhibitors such as tissue inhibitors of metalloproteinases (TIMPs) [19]. Four TIMPs can reversibly block MMP activity. TIMPs are proteins of 21–28 kDa which bind the active site of MMPs in a one-to-one ratio [7, 20]. Besides TIMPs, also *α*-2 macroglobulin and receptor mediated endocytosis can prevent activated MMPs from exerting their effects. MMPs can be activated by different stimuli. Proinflammatory cytokines (e.g., TNF*α* and IL-1*β*) and several growth factors can initiate an intracellular signaling cascade leading to the activation of AP-1, NF-kB, or ETS transcription factors, with consequent MMP transcription [21]. On the level of MMP-zymogen activation, mostly serine proteases and other MMPs are involved in initiating proteolytic cleavage of the prodomain; for example, MMP-3 can cleave pro-MMP-9 [22]. Additionally, high levels of reactive oxygen species (ROS) and reactive nitrogen species can induce activation of MMPs [23]. It has been observed that MMPs in the CNS are secreted by microglia, astrocytes, and neurons [24]. In physiological conditions, MMPs are either absent or present at undetectable levels in the mature brain, and deregulation of their activity could shift the balance and induce perpetuation of chronic inflammation. This has been shown in different peripheral chronic diseases, such as atherosclerosis and rheumatoid arthritis [25, 26], and neuroinflammatory diseases, such as cerebral ischemia, stroke, and bacterial meningitis [27]. MMPs are known to be involved in CNS barrier maintenance and to increase the permeability of the barriers in inflammation. The proposed mechanism involves degradation of ECM components (e.g., laminin and collagen), which normally support cellular structures and limit the passage of different molecules and cells into the blood through the barriers [28–30]. Additionally, increased MMP activity is known to negatively affect tight junction functionality at the CNS barriers [30–33]. Alternatively, several MMPs have been shown to activate proinflammatory cytokines and free radicals, which enhances inflammation and subsequently induces disruption of the CNS barriers [27, 34, 35]. MMPs are secreted upon inflammation, for example, by peripheral neutrophils, which thereby contribute to aggravation of the inflammation and disruption of the barriers [36]. Compromise of CNS barrier integrity due to activation of MMPs has been observed in cerebral ischemia, traumatic brain injury (TBI), and other diseases associated with neuroinflammation [37]. In this review, we focus on the role of different MMPs in the major neurodegenerative diseases, AD, PD, ALS, HD, and MS (Figure 2; Tables 2 and 3).

## 3. Neurodegenerative Diseases and Neuroinflammation

### 3.1. Neurodegenerative Diseases

During the previous century, prolongation of the human lifespan led to an increase in the proportion of elderly people in the population, which has given rise to an increased incidence of age-related diseases such as neurodegenerative disorders. Neurodegenerative diseases share common features, such as progressive loss of neurons and deterioration of the structure and function of the central and/or peripheral nervous system. These chronic illnesses impose a heavy economic and social burden and affect both patients and caregivers. Since all neurodegenerative diseases are incurable, the outcome in most cases is death. AD represents 60–70% of all neurodegenerative cases [38]. Worldwide, 47 million people are living with AD and other dementias, including more than 5 million in the United States alone [39], where the number is expected to rise to 7.1 million in 2025 [40]. The estimated costs of medical care exceed 214 billion dollars annually [39]. Furthermore, there are large numbers of patients with other neurodegenerative diseases, including AD, PD, ALS, HD, MS, and frontotemporal dementia [41].

The major problem in the management of neurodegenerative diseases is the lack of adequate information on their pathogenesis and absence of mechanism-based treatments. However, interaction of genetic and environmental factors, as well as advanced age, is known to contribute to disease onset. Generally, all neurodegenerative diseases share certain molecular and cellular mechanisms, including protein aggregation and formation of inclusion bodies.

The traditional classification of neurodegenerative disorders is based on clinicopathological features and has been formulated through “consensus criteria,” that is, a set of diagnostic criteria on which experts in the field agree, along with specific molecular characteristics. This traditional classification was established during the early stages of the research on neurodegenerative diseases and was based on a rather small number of cases [96]. One of the categories used for classification is the presence of certain molecules in brain lesions associated with the disorder, for example, *β*-amyloid (A*β*) for AD and *α*-synuclein for PD. For several neurodegenerative diseases, mutations in specific genes have been found, for example, presenilin genes (PSEN 1 and 2) for AD. However, in certain cases diagnosis is difficult due to the coincidence of clinicopathological features of several neurodegenerative diseases [97–99]. Nowadays, with the increasing amount of molecular and genetic data and the continuing efforts to find other common characteristics of neurodegenerative diseases, questions have arisen about the distinction between these diseases, making their classification even more problematic [99–101]. The early classifications divided neurodegenerative diseases into either taupathies (including AD, Pick's disease (PiD), argyrophilic grain disease (AGD), progressive supranuclear palsy (PSP), corticobasal degeneration (CBD), and FTDP-17) and synucleinopathies (including PD, dementia with Lewy bodies (DLB), and multiple system atrophy (MSA)) [102]. Today, different, nosological classifications of neurodegenerative diseases exist, taking into account clinical presentation, affected brain regions and cell types, altered proteins involved in the pathogenesis of the disease, genetics, and possible overlaps between diseases [96, 103].

### 3.2. Neuroinflammation

Neuroinflammation is defined as inflammation of nervous tissue, which occurs as a biological response to different signals, such as infection, CNS injury, autoimmunity, and toxic compounds. Although it is initiated primarily as a beneficial reaction of the CNS to the harmful stimulus, it could eventually aggravate the disease. Neuroinflammation is characterized by the initiation of a cascade of events, including production of cytokines and chemokines accompanied by the release of free radicals and proteases, resulting in a chronic inflammatory state in the organism.

Four CNS barriers separate blood from CNS parenchyma to block entrance of immune cells and various molecules into the brain from the periphery: blood-brain barrier (BBB), blood-cerebrospinal fluid barrier (BCSFB), arachnoid barrier, and blood-spinal cord barrier (BSCB) [104, 105]. All CNS barriers are dynamic structures that allow passage of immune-related molecules and cells upon specific stimuli (e.g., proinflammatory cytokines) [106, 107]. Furthermore, immune functions in the brain are conveyed by microglia, innate immune cells that are constitutively present in the brain, but astrocytes can secrete many molecular mediators of the immune response [108]. Microglia are of myeloid origin and normally reside in a ramified, resting state in the CNS, where they monitor the surrounding environment in the brain and spinal cord. Opposite to immune cells in the periphery, microglia are predominantly involved in limiting inflammation. When triggered by various immunological stimuli, microglia morphologically transform into ameboid cells and start to proliferate and secrete inflammatory mediators [109, 110]. Besides, they start to mimic antigen presenting cells (APC), which are immune cells in the periphery, by upregulating major histocompatibility complex (MHC) class II and becoming phagocytic [111]. The presence of the MHC is important for prolongation of the immune response in the CNS. Activated microglia engage in different processes, both protective and harmful. It has been shown that microglia are involved in neuron survival [112], neurogenesis [113], facilitation of brain repair* via* guidance of stem-cell migration to the site of inflammation or injury [114], and clearance of cell debris [115]. Nevertheless, when overactive, microglia can inflict severe damage to the brain by excessive production of molecules such as MMPs, proinflammatory cytokines and chemokines, and cytotoxic molecules such as ROS and nitric oxide (NO) [116–118]. Astrocytes too play a role in the inflammatory response of the CNS. They also produce different inflammatory mediators, including cytokines, chemokines, and complement components [119]. For example, both microglia and astrocytes secrete IL-2 and IL-1*β*, thereby stimulating CD4+ T helper cells to produce GM-CSF, which contributes to perpetuation of the inflammation processes by recruitment of CD11b-positive myeloid cells [120]. Also T cells can secrete cytokines and MMPs that are known to disrupt the BBB and permit the entry of immune cells from periphery, converting acute inflammation into a chronic inflammatory state. Although microglia have been implicated in neurodegenerative diseases, the mechanisms responsible for activating microglia are unknown [121].

## 4. Role of MMPs in Neurodegenerative Diseases

### 4.1. Aging

All neurodegenerative diseases are multifactorial and caused by complex interactions of genetic and environmental factors. By far the most prominent risk factor for most neurodegenerative diseases is aging, so the prevalence of these disorders is high above the age of 65 years. Aging is influenced by interaction and balance between various protective and harmful factors over the lifespan of an individual. These factors include genetics, nutrition, psychosocial influences, and exposure to toxic compounds [122]. However, it is not yet clear how aging acts on the development of neurodegenerative diseases and other age-related diseases [123]. Two of the most prominent characteristics of aging are immunosenescence (deterioration of immune responses) and inflammaging (presence of chronic low grade inflammation) [124, 125]. Physiological aging is associated with a progressive increase in the number of activated microglial cells in the brain and spinal cord [126–128] accompanied by transition from normal microglial morphology to microglial dystrophy [129]. It has been shown that aged microglia secrete IL-1*β* [130] and increase MHC class II expression [131]. Low-level systemic inflammation during aging has also been linked to the presence of active microglia and increased proinflammatory cytokines levels in the brain [132–134]. One of the characteristics of aging is accumulation of advanced glycation end products (AGE), which are also elevated in AD. Activation of the receptor for AGE (RAGE) leads to release of proinflammatory cytokines and free radicals, further contributing to inflammatory processes [135]. Data are scarce on the role of MMPs in brain aging. Nevertheless, changes in the physiological balance between MMPs and TIMPs have been related to age-related vascular diseases [136]. One theory of aging suggests that vascular-derived insults initiate and/or contribute to aging and to some of the age-related diseases, such as AD [137, 138]. In a comparative study, RNA expression of 22,626 genes was monitored in the heart and cerebellum of young and aged mice of several strains. Two potential biomarkers of aging present in both structures were identified: complement component C4 and TIMP-2 [139]. Using magnetic resonance imaging, Romero et al. found that upregulation of MMP-9 was associated with aging and circulating levels of MMP-9 and TIMP-1 in patients with brain ischemia and aging [140]. Liu et al. observed that MMP-12 increased in the aging brain and that MMP-12 deficiency led to a reduction of neuroinflammaging. This finding is linked to aggravation of neuroinflammation associated with aging* via* the induction of the migration of bone marrow derived microglia to the brain [141]. Furthermore, Safciuc et al. showed that microvessels in the brain of aged rats exhibit decreased MMP-2 activity and appearance of MMP-9 [142].

### 4.2. Alzheimer's Disease (AD)

AD is the most common neurodegenerative disorder. Its prominent characteristics include brain atrophy, caused by neuronal cell death, and decreased dendritic arborization in the cerebral cortex and other subcortical areas. The hallmarks of AD include presence of amyloid plaques and neurofibrillary tangles, which are linked to cerebral atrophy [143]. Amyloid plaques, also called senile plaques, appear in brain parenchyma as extracellular deposits consisting of A*β* fibrils of 37–43 amino acids, originating from alternative processing of APP protein [144]. Although A*β* deposition is considered a signature lesion for AD, it also occurs in Down's syndrome, possibly due to triple multiplication of amyloid precursor protein (APP), as well as in certain cases of dementia with Lewy bodies [145, 146]. Additionally, occurrence of A*β* pathology has been observed in several other neurodegenerative diseases, such as PD, Pick's disease (PiD), progressive supranuclear palsy (PSP), and corticobasal degeneration [147], as well as in ALS [148]. Besides, deposition of A*β* occurs in the cerebral vasculature in both cerebral amyloid angiopathy (CAA) and in 90% of patients with AD [149]. A*β* originates from the transmembrane protein APP, which undergoes amyloidogenic processing by *β*-secretase to produce *β*-C terminal fragments (CTFs). These fragments are cleaved by *γ*-secretase to release A*β* in the extracellular space and APP intracellular domain (AICD) into the cytoplasm. In the physiological nonamyloidogenic pathway, *α*-secretase cleaves APP at a different site, producing *α*-CTFs. These fragments are cleaved by *γ*-secretase, resulting in the cytoplasmic peptide fragment AICD and the extracellular p3 peptide. In contrast to A*β* peptides, p3 peptides have a low propensity to assemble into stable oligomers and they have no known harmful effects on brain cells [150]. It has been shown that some of the members of the metalloproteinase family, including the ADAM (a disintegrin and metalloproteinase) proteins ADAM-17 (also called tumor necrosis factor-*α*-converting enzyme or TACE), ADAM-9 and ADAM-10, can cleave APP at the *α*-secretase cleaving site [150]. Released A*β* can act as a monomer, but it can dimerize or oligomerize. A*β*-induced synaptic dysfunction has been noted in AD [151–154]. A*β* oligomers are nonfibrillar *β* structures, and further aggregation of A*β* oligomers results in the formation of protofibrils and eventually fibrils. These structures form a base for formation of A*β* plaques [155–157]. A*β* deposits are typically surrounded by dystrophic neurites, reactive astrocytes, and activated microglia, forming dense-core plaques in the brain parenchyma [158].

Also considered hallmarks of AD are neurofibrillary tangles (NFT), intercellular deposits of a hyperphosphorylated form of Tau protein [159]. Physiologically, Tau protein plays a role in the assembly and stabilization of microtubules in neurons [160]. Cases of dementia with abundance of NFTs and a few amyloid plaques have been classified as a nonspecific type of neurodegenerative disorders, called NFT dementia [161]. However, the etiology of AD is complex, and neither A*β* nor NFT alone should be considered responsible for the disease manifestations. Therefore, other proposed mechanisms and manifestations of the disease should also be taken into account [162]. Some authors point to the role of an imbalance in ROS formation and cellular antioxidant activity in AD [163]. The idea is that overproduction of free radicals could be a driving force behind neurodegeneration, and given the large number of possible stressors, such as aging, inflammation, hypoxia, and cerebral hypoperfusion, there is ample opportunity for overproduction of free radicals [164]. On the other hand, advocates of the inflammatory hypothesis propose a central role for activated microglia nearby amyloid plaques in the brain. Their notion is that activated microglia produce large amounts of inflammatory cytokines and chemokines, which sustain a chronic inflammation in the brain that ultimately leads to neuronal cell death [165]. Furthermore, it has been shown that besides the established direct neurotoxic effect [166], A*β* can exert indirect proinflammatory effects* via* microglial activation, which results in secretion of NO, TNF*α*, and superoxides [167, 168]. Interestingly, clustering of activated microglia around A*β* aggregates has been observed even before the development of AD symptoms [169–171].

**Figure 3:**
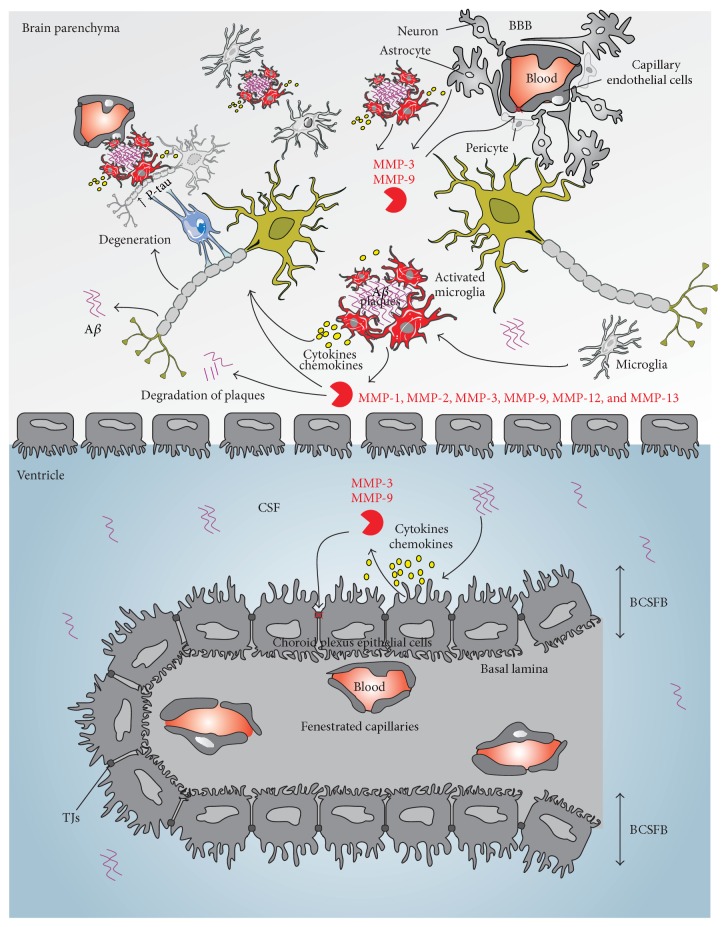
Schematic representation of the involvement of MMPs in Alzheimer's disease pathology. A*β* peptides produced from APP processing form oligomers that subsequently form amyloid deposits or plaques in the brain parenchyma. A*β* oligomers activate inflammatory cells in the brain (astrocytes, microglia, and choroid plexus epithelium). Once activated, microglia change their shape, migrate close to plaques, and begin to secrete proinflammatory cytokines and MMPs. Secreted MMPs degrade A*β* and, on the other hand, exacerbate inflammation in the brain, leading to death of neurons. These cytokines and MMPs also affect the endothelial tight junctions, alter the pericyte phenotypes, and contribute to increased BBB permeability. Similarly, oligomers in the CSF activate the choroid plexus epithelium, which leads to the release of proinflammatory cytokines and MMPs. These secreted MMPs further damage the tight junctions at the BCSFB. A*β*, *β*-amyloid; BBB, blood-brain barrier; BCSFB, blood-cerebrospinal fluid barrier; CSF, cerebrospinal fluid; MMP, matrix metalloproteinase; TJs, tight junctions.

In view of the relationship between MMPs and AD [62] (Figure 3) and in order to distinguish AD from vascular dementia, Bjerke et al. proposed MMP-9 and TIMP-1 as biomarkers of AD, next to T-tau, P-tau, A*β*_1–42_, and white matter lesions [52]. Strikingly, a correlation between cognitive impairment and MMP-9 activity was observed in patients with mild cognitive impairment [53]. In agreement with that correlation, Lorenzl et al. observed higher levels of MMP-9 in serum of AD patients [54]. MMP-9 expression was shown to be induced in AD patients in neuronal cytoplasm, neurofibrillary tangles, amyloid plaques, and vascular tissue [56], as well as in astrocytes upon A*β* stimulation [57]. Additionally, MMP-9 was found in pyramidal neurons of the brains of AD patients, and near amyloid plaques and it was shown that MMP-9 is able to cleave A*β*_1–40_ [58]. Moreover, Yan et al. showed that MMP-9 can degrade A*β* fibrils* in vitro*, as well as amyloid plaques in brain slices from APP/PS1 mice [55]. Using intracerebroventricular (icv) injections of different A*β* peptides in animal models, Mizoguchi et al. showed an increase in MMP-9 activity in hippocampus, related this increase to A*β*-induced cognitive impairment, and confirmed the results using MMP inhibitors and MMP-9 knockout mice [59]. Notably, another study showed that MMP-3, by remodeling the ECM, is crucial for synaptic plasticity and learning [48]. It has been shown that MMP-9 can act through NMDA receptor signaling* via* an integrin *β*1 dependent pathway [60]. Li et al. showed in primary astrocyte cultures insignificant levels of MMP-9 in medium after treatment with A*β* oligomers, accompanied with a decrease in MMP-2 activity. On the contrary, in the brain of APP/PS1 AD mice, they observed increased MMP-2 and proinflammatory cytokine levels. They proposed that A*β* can decrease the expression and activation of MMP-2 in astrocytes directly, while stimulating microglia to produce proinflammatory cytokines, which in turn again induce MMP-2 expression and aggravate the disease [43]. However, in the study of Bruno et al., no elevation of MMP-2 activity was observed in AD patients [53]. In a transgenic mouse model of AD, expression of MMP-2 and MT1-MMP, a potent MMP-2 activator, was found in reactive astrocytes around amyloid plaques [45], and higher levels of A*β*_1–42_ increased the production of MMP-3, MMP-12, and MMP-13 in microglia [47]. Additionally, MMP-12 exacerbates the cascade of proteolytic processes by subsequent activation of other MMPs such as MMP-2 and MMP-3 [47]. Kook et al. looked into the effects of A*β* on endothelial cells and BBB integrity and linked this to MMP activity. They observed increased BBB permeability in cultured endothelial cells linked to decreased zonula occludens-1 (ZO1) levels, one of the major components of TJs. Moreover, there was an increase in MMP-9 and MMP-2 activity, and broad-spectrum MMP inhibition reversed the A*β*-induced BBB disruption. Additionally, they confirmed these results in a transgenic mouse model of AD by showing enhanced immunoreactivity of MMP-9 near cerebral capillaries and alterations in tight junction components. The proposed mechanism of A*β* activity is through activation of RAGE, which is physiologically expressed on endothelial cells and activates the intracellular calcineurin (CaN) signaling pathway, which ultimately results in activation of MMPs and TJ cleavage [172]. In the same year, another group showed that interaction of A*β* with RAGE induces MMP-2* via* the ERK and JNK pathways in brain endothelial cells [44].

Besides aging, the most prominent genetic risk factor for developing late onset AD is the presence of apolipoprotein E *ε*4 allele (APOE *ε*4) in the genome [173]. The group of Zlokovic reported that, both in transgenic mice and in humans, APOE *ε*4 leads to BBB breakdown by activating the proinflammatory cyclophilin A (CypA)/MMP-9 pathway in brain pericytes, which are important components of the neurovascular unit and guardians of BBB integrity [61, 174]. This eventually results in degradation of the BBB tight junctions and basement membrane proteins [61, 174].

As far as the role of stromelysins in AD is concerned, MMP-3 levels were significantly upregulated in plasma, similar to what was observed in CSF of AD patients [49]. In contrast, Mlekusch and Humpel observed downregulation of MMP-3 and MMP-2 in CSF of AD patients, but it should be noted that these patients had lower A*β* levels [175]. In other studies, it has been shown that MMP-3 is expressed in microglia, astrocytes, and endothelial cells in the brain, as well as near senile plaques in AD [62]. Deb and Gottschall showed that MMP-3 was induced and its activity increased in astrocyte and neuronal cell cultures upon A*β*_1–40_ stimulation [46]. As reported for MMP-9, MMP-3 can degrade A*β* [47, 50, 176]. Moreover, a correlation was found between MMP3*∗*5A and APOE 4 alleles, and the presence of both is a risk factor for developing AD [177]. Interestingly, we recently reported that icv injection of A*β*_1–42_ oligomers induces loss of barrier integrity at the blood-CSF barrier, and this was linked to increased MMP-3 expression and MMP activity [32]. Moreover, the A*β*_1–42_ oligomer-induced leakage of the BCSFB could be prevented by a broad-spectrum MMP inhibitor and did not occur in MMP-3 deficient mice [32].

Other MMPs have been implicated in AD. Leake et al. reported a notable increase in MMP-1 in brain of AD patients [42]. Langenfurth et al. found upregulated microglial/macrophage expression in tissues from AD patients, as well as in a mouse model of AD [178]. Finally, levels of TIMP-1 and C-reactive protein (CRP) were found to be increased in AD patients, and they decreased remarkably after treatment with acetylcholinesterase inhibitors (AchEIs), one of the few available therapies of AD [179].

### 4.3. Parkinson's Disease (PD)

PD is the second most prevalent neurodegenerative disease and the most common neurodegenerative movement disorder, with an estimated 7–10 million people worldwide suffering from it. Like AD, its prevalence increases with age, and due to the severity and long duration of the disease, its costs in the US alone are estimated at 25 billion dollars per year. A prominent characteristic of PD is the presence of intracellular protein inclusions called Lewy bodies in affected brain areas. These inclusions are formed of fibrillar, misfolded proteins composed of *α*-synuclein, parkin, synphilin, synaptic vesicle proteins, and neurofilaments. The biological significance of these inclusions is unclear [180]. Interestingly, inclusions similar to Lewy bodies were found in 22% of cases of familial AD, and they occur also in dementia with Lewy bodies (DLB) and in multiple system atrophy (MSA) [181]. Another hallmark of PD is selective and progressive loss of dopaminergic neurons in substantia nigra pars compacta. The substantia nigra, being part of basal ganglia together with the striatum, globus pallidus, and subthalamic nucleus, modulates motor activity in the brain [182]. Thus, due to dopaminergic neuron cell death, one of the clear manifestations of the disease is loss of control over movements, resting tremor, bradykinesia, and rigidity [183]. Additionally, PD is accompanied by sensory dysfunction, mood and sleep disorders, dementia, and partial autonomic nervous system impairment [184]. This multifactorial disorder is known to be influenced by genetic factors, such as multiplication or missense mutations in the *α*-synuclein gene, which is a major risk factor for familial PD [185]. Additionally, mutations in parkin, PINK1, and LRRK2 have been identified as possible causes of other cases of familial PD [186, 187]. Nevertheless, most cases of PD are sporadic, and the onset of the disease has been shown to be caused by complex interaction of environmental and genetic factors. PD is characterized by chronic inflammation persisting over the years of disease progression. Thus, some hypotheses propose that neuroinflammation could play a pivotal role in promotion and aggravation of the disease [188–190]. Studies on postmortem tissue of PD patients, as well as* in vivo* imaging, revealed astrogliosis, overactivation of microglia, and infiltration of peripheral immune cells into brain regions affected by PD [191–195]. In PD patients, active microglia have been observed in most of the regions with Lewy bodies, and* in vivo* imaging revealed microglial activation throughout the different stages of the disease, indicating chronic microglial activation [196]. The substantia nigra seems to be particularly susceptible to inflammation due to the presence of the largest number of microglia in this brain area, so relevant stimuli lead to the activation of large numbers of microglia [197]. In agreement with these findings, activated microglia have been found in substantia nigra in patients with sporadic or familial PD [196, 198] as well as after exposure of humans to 1-methyl-4-phenyl-1,2,3,6-tetrahydropyridine (MPTP) [65]. The same effect was seen in the substantia nigra and striatum in animal models of PD based on MPTP injection [199–202]. Microglial activation was also found in other PD models [203] and in other brain regions in PD patients, including putamen, hippocampus, transentorhinal cortex, cingulate cortex, and temporal cortex [204]. Hirsch and Hunot described early microglial activation after MPTP injection, followed by later neuronal cell death and infiltration of T cells and astrogliosis [189]. Other evidence for the role of microglia in PD comes from studies using anti-inflammatory drugs to inhibit microglial activation, which was protective against neurodegeneration induced by MPTP or 6-OHDA [205]. Active microglia are known to secrete inflammatory mediators, and accordingly, increased proinflammatory cytokine levels were increased in the substantia nigra [206, 207] and CSF in PD [208]. Furthermore, IL-1*β* and IL-6 were found to be elevated in CSF of Parkinson's disease patients [209, 210].

It has been speculated that prolonged overactivation of microglia and production of proinflammatory cytokines could lead to neuronal degeneration in PD [207, 211]. It is also speculated that oxidative stress could be generated from dopaminergic metabolism, mitochondrial dysfunction, and microglial activation, which could be influenced beforehand by various toxins and mutations in Parkin, PINK1, DJ-1, or HtrA1, which are important for physiological mitochondrial functioning [212]. Other evidence for the involvement of active microglia in triggering neurodegeneration of dopaminergic neurons in the substantia nigra comes from studies on injection of lipopolysaccharide (LPS) systemically or directly in the substantia nigra [213–215]. Intriguingly, GABAergic and serotonergic neurons remained unharmed, while dopaminergic neurons were lost [216]. This could be explained by specific susceptibility of dopaminergic neurons to oxidative stress due to the presence of tyrosine hydroxylase and monoamine oxidase, which are ROS-generating enzymes, as well as excessive production of easily oxidized cytosolic dopamine [217, 218]. The mechanism of microglial activation remains unclear. However, it is speculated that initial neurotoxic insult to dopaminergic neurons results in the release of certain factors that activate microglia and convert them from beneficial into harmful [121]. It has been shown that damaged dopaminergic neurons can activate microglia by releasing *α*-synuclein [219] and neuromelanin, causing them to produce ROS [220].

MMP-3, produced by neurotoxin-stressed dopaminergic neurons, seems to be a self-sufficient player in microglial activation in the absence of any other inflammatory molecule. It has been suggested that this mechanism plays an important role in apoptosis. On the one hand, MMP-3 could cleave the connections between apoptotic cells and ECM, thereby facilitating subsequent phagocytosis. On the other hand, it could activate microglia, leading to the release of cytokines and receptors for phagocytosis of apoptotic cells [64]. In an* in vitro* study, Kim et al. observed that the ERK signaling pathway is induced in microglia after MMP-3 stimulation. Additionally, they hypothesized that both active MMP-3 and catalytically active recombinant MMP-3 could activate microglia to produce proinflammatory cytokines, which in turn aggravate neuronal apoptosis of damaged cells, leading to further induction of apoptosis in neighboring dopaminergic neurons. This hypothesis is supported by postmortem studies describing progressive dopaminergic neuronal degeneration in humans and monkeys treated with MPTP for 10 years [65, 221]. Using MMP-3 deficient mice and a broad spectrum MMP inhibitor, it has been shown that depletion of MMP-3 can significantly reduce MPTP-induced degeneration of nigrostriatal dopaminergic neurons in the brain [66]. Furthermore, MMP-3 activated microglia produce superoxide, known to be involved in facilitation of dopaminergic neuronal cell death* in vitro* [222, 223] and* in vivo* [224]. Using siRNA, another group confirmed that MMP-3 is actively secreted by neurons [51]. Also upregulated MMP-9 activity, produced by neurons and microglia, was found in both striatum and substantia nigra after MPTP treatment, and pharmacologic inhibition of MMPs protected against MPTP neurotoxicity [69]. Earlier, the same group analyzed postmortem brain tissue from PD patients and found no change in the activities of MMP-9 and MMP-1 in substantia nigra, cortex, or hippocampus, whereas MMP-2 was significantly reduced in the substantia nigra. Additionally, they showed that MMP-9 was localized primarily in neurons and MMP-2 in astrocytes and microglia. In the same study, TIMP-2 levels did not change, whereas TIMP-1 was upregulated in substantia nigra but not in the cortex and hippocampus [63]. The increase in MMP-9 expression in substantia nigra was later confirmed by Annese et al., who also demonstrated MMP-9 expression in striatum. The data showed that MMP-9 is expressed in reactive microglia and astrocytes, pinpointing MMP-9 as a key molecule for the onset of neuroinflammation in PD. Experiments done in MMP-9 deficient mice confirm that active glia diminish neuronal survival since decreased numbers of active microglia correlated with increased numbers of functional dopaminergic neurons [70]. In a primate model of PD (MPTP-injected macaques), an increase in MMP-9 labeled striatal neurons and astrocytes was also found.

BBB leakage was found in animal models of PD solely in brain regions and was associated with microglial activation and dopaminergic neurodegeneration [225, 226]. Recent evidence suggests that the proteolytic activity of MMPs might be involved in alteration of *α*-synuclein protein conformation, thus contributing to aggregation, Lewy body formation, and microglial activation [227]. In an* in vitro* study on a dopaminergic neuronal cell line, Sung et al. observed MMP-dependent proteolysis of *α*-synuclein, followed by increased aggegate formation. In this process, MMP-3 was particularly efficient, but MMP-1, MMP-2, and MMP-14 showed similar properties [68]. Levin et al. further studied the MMP-specific *α*-synuclein cleavage and showed that both MMP-1 and MMP-3 mediate increased *α*-synuclein aggregation in comparison to trypsin and proteinase K [228].

### 4.4. Amyotrophic Lateral Sclerosis (ALS)

ALS, also known as Lou Gehrig's disease, is characterized by degeneration of motor neurons in the brain, brainstem, and spinal cord. All voluntary muscles are affected, and the muscle weakness and atrophy are followed by paralysis, and finally respiratory failure and death [36]. Some other neurodegenerative diseases share similar etiology, such as progressive lateral sclerosis (PLS), progressive muscular atrophy (PMA), ALS dementia, and ALS frontal lobe dementia [229]. Interestingly, one-third of all ALS patients exhibit symptoms or pathology resembling those of AD [148]. The incidence of the disease is relatively rare (2.08 people per 100,000 in Europe), and the prevalence is mostly in people between the ages of 45 and 65 years [230]. Familial occurrence of the disease is only 5–10% of all ALS cases, and the cause of the sporadic form of ALS is still unknown. Some familial and sporadic cases are caused by mutation in the gene for copper-zinc superoxide dismutase 1 (SOD1) [231]. Additionally, ALS is associated with protein inclusions composed mostly of transactive response DNA-binding protein 43 (TDP-43) in the cytoplasm in the affected areas of the brain and spinal cord [232]. However, occurrence of TDP-43 is not characteristic of only ALS but was found in several patients with frontotemporal lobar degeneration with TDP proteinopathy (FTLD-TDP), as well as in frontotemporal dementia, AD, and some other neurodegenerative diseases [96]. The etiology of the disease is unknown, but various mechanisms have been proposed, including neuroinflammation, glutamate excitotoxicity, oxidative stress damage and mitochondrial dysfunction, protein misfolding and aggregation, and deficits in neurotrophic factors [233]. Besides, in ALS, active microglia were also observed in brain areas such as motor cortex, pons, dorsolateral prefrontal cortex, and thalamus. Interestingly, activation of microglia was correlated with progression of the disease [234]. In the* in vitro* work of Swarup et al., microglia overexpressing TDP-43 increased their secretion of proinflammatory cytokines upon LPS treatment in comparison to wild type microglia [235]. One of the theories suggests that BBB and BSCB breakdown could contribute to the motor neuron damage, due to the importance of these barriers in maintenance of homeostasis in the CNS. Indications of the involvement of MMPs, key players in barrier alteration, come from early studies on neocortex and spinal cord of ALS patients, in which MMP-2 was found in astrocytes, and MMP-9 was found in pyramidal neurons in the motor cortex and motor neurons in the spinal cord. Additionally, MMP-2 activity was decreased in motor cortex whereas MMP-9 activity was increased in spinal cord [236]. Since BSCB disruption in ALS [237–239] is accompanied by downregulation of mRNA for tight junction proteins [240], Miyazaki et al. speculated that MMP-9 is involved in barrier disruption [241]. Another group showed reduced MMP-9 activity during disease progression, with the peak at the onset of ALS, and described a similar profile for MMP-2 [74]. Two separate groups found significant increases in both pro-MMP-9 and active MMP-9 in serum of ALS patients relative to healthy controls [242, 243]. Niebroj-Dobosz et al. reported that in mild cases of ALS, expressions of MT-MMP-1, MMP-2, MMP-9, and TIMP-1 are elevated in serum compared to CSF, where MT-MMP-1, MMP-2, and TIMP-1 were upregulated or unchanged while MMP-9 levels were decreased [75]. Furthermore, Fang et al. found increased levels of MMP-9 in CSF from patients suffering from rapidly progressing ALS. They speculated that this finding is associated with progression of the disease, poor survival of the patients, and neuronal degeneration. Nevertheless, MMP-2 showed a slow but progressive decrease with the development of the disease [71]. In the study by Kaplan et al., diminishing MMP-9 function by genetic, viral, or pharmacological intervention was shown to prolong survival in a SOD1 mouse model of ALS [244]. Moreover, MMP-9 was preexpressed only in fast motor neurons, which have been shown to be particularly susceptible to degeneration in patients suffering from ALS. These results show that MMP-9 is a key player in the onset of the disease and point to it as a therapeutic target.

Kaplan et al. focused on the early stage of the disease and expression of MMP-9 by neurons, whereas Kiaei et al. studied later stages of the disease and observed expression of MMP-9 by activated microglia, giving support to the hypothesis that the pathology is mediated by cytokines secreted by microglia. Since the depletion of MMP-9 gene does not rescue transgenic SOD1 mice from death, ALS indeed has complex background [73].

### 4.5. Multiple Sclerosis (MS)

MS is a chronic, autoimmune, and inflammatory disease of the CNS. The hallmarks of the disease are demyelinated areas, with moderate preservation of axons. There are about 2.5 million cases worldwide, with approximately 400,000 in the US alone, and MS is twice as common in women as in men [245]. In contrast to most neurodegenerative disorders that are prevalent in aged individuals, MS occurs in people between 20 and 45 years of age [246]. The cause of the disease is unknown, but genetic and environmental factors contribute to its development. Interestingly, epidemiological studies revealed a correlation with smoking, exposure to UVB radiation, and intake of unsaturated fatty acids [247]. Four major categories of MS exist. (1) Relapsing-remitting MS (RRMS) occurs in about 85% of patients suffering from MS. The disease alternates between remission (periods of improvement) and relapses (periods of deterioration). (2) Secondary progressive MS (SPMS), which is characterized by continuous worsening of the symptoms, affects some patients suffering from RRMS. (3) Primary progressive MS (PPMS) is manifested in about 10% of MS patients. This group shows constant aggravation of the disease with no remissions or relapses. (4) Progressive-relapsing MS (PRMS) is the rarest type, present in less than 5% of patients. Although it is progressive from the beginning, it shows relapses occasionally, but without periods of remission.

At onset, RRMS is characterized as a neuroinflammatory state [248]. However, with the progression of the disease and occurrence of relapses, certain residual disability develops. Over ten years, most patients enter SPMS, which is then observed more as neurodegeneration state resulting in permanent disability [249].

Although inflammation is considered as primary in MS, this disease is recently being acknowledged as a neurodegenerative disorder too because of recent findings. It has been observed that disability related to MS is correlated with axonal damage and neuronal cell loss more than with inflammation. The new hypothesis that emerged resembles the previously proposed mechanism for the other neurodegenerative disorders: perpetuated inflammation leads to triggering of neurodegenerative processes [250]. Interestingly, certain case reports have noted patients suffering from MS and ALS at the same time [251].

In MS, the BBB is disrupted, leading to peripheral blood leukocyte infiltration, followed by focal degradation of myelin, and finally axonal disruption and neuronal cell loss. Data show involvement of MMPs in each of these processes [252–255]. It has been shown that the BBB function in MS is lost in both relapsing-remitting and progressive phases. Nevertheless, BBB dysfunction is a temporary event, although recurrence is highly possible [256]. Functional changes in the BBB were observed in postmortem brains of MS patients [257]. In fact, local BBB changes that follow the pattern of the lesions help diagnose MS by observing brains using magnetic resonance imaging (MRI) with contrast agents that easily leak into the affected parts of the brain [258]. Some of the data suggest that BBB breakdown precedes infiltration of the immune cells, but this event is not definitively the primary cause of lesion formation [258]. Morgan et al. showed in a common animal model of MS called experimental autoimmune encephalomyelitis (EAE) that occludin dephosphorylation preceded visible signs of disease onset, which indicates that BBB breakdown is one of the first events in MS [259]. Alterations in tight and adherent junction morphology in MS have also been described [258].

It is known that in MS, various brain and immune cells can secrete MMPs, thus contributing to the BBB breakdown [260, 261]. Cossins et al. observed expression of MMP-7 by macrophages and MMP-9 in blood vessels in active lesion sites of postmortem brain samples [82]. Another group confirmed this finding and also showed expression of MMP-3 in endothelial cells, MMP-1, MMP-2, MMP-3 and MMP-9 in macrophages, and to a lesser extent in astrocytes, around active and necrotic lesions [80].

Lepert et al. examined CSF samples from patients suffering from both RRMS and PPMS and found an increase in MMP-9 in all the RRMS cases throughout both phases of the disease. However, in PPMS patients, MMP-9 was increased in only about half of the samples and in significantly smaller amounts than in the relapsing-remitting form. They argued that this points out that T-cells and macrophages are responsible for the secretion of MMP-9 in MS. Additionally, they proposed that constant elevation of MMP-9 throughout the progress of the disease could contribute to the surrounding tissue damage and neuronal cell loss [83]. In their work using the EAE model, Kieseier et al. showed that the increase in MMP-9 and MMP-7 expression in blood vessels and parenchyma strongly correlated with the peak of the disease [262].

Elevated levels of MMP-9 were also observed in serum of MS patients, together with an increase in TIMP-1 and TIMP-2. In the same study [84], the authors pointed out an association of these increases with the number of lesions observed by MRI. However, the study of Waubant et al. found increased levels of MMP-9 in serum but no elevation of TIMP-1 levels. Also, by univariant analysis they found that an increase in MMP-9 and a decrease in TIMP-1 levels preceded the appearance of new lesions [263]. Another group compared the mRNA levels of MMP-1, MMP-3, MMP-7, MMP-9, MMP-14, and TIMP-1 in blood monocytes of MS patients with those of controls. They found that all except MMP-14 were upregulated [81]. In an interesting study by Althoff et al. using the EAE model, transgenic mice that constitutively express TIMP-1 in the CNS had a normal phenotype but EAE symptoms were diminished [264]. Interestingly, other studies done using the EAE model showed limited BBB restoration and amelioration of the clinical picture after administration of broad-spectrum MMP inhibitors [90, 265]. Finally, MMP-9 knockout mice were shown to be less susceptible to induction of EAE [266]. Besides the occurrence of the leakage, the BBB also becomes activated, meaning that cells making up the BBB, including endothelial cells, astrocytes, and potentially pericytes, start expressing and secreting various factors involved in the recruitment and functioning of leukocytes [258]. Constant leukocyte migration occurs through the BBB in active MS lesions and this migration is normally strictly regulated by a number of molecules, such as cell adhesion molecules (CAM), integrins, cytokines, and chemokines. The leukocyte infiltration further aggravates BBB breakdown, as shown in* in vitro* studies [267]. In one* in vitro* study, interferon *β* treatment downregulated MMP-9 expression and abolished MMP-2 expression, thereby diminishing subsequent migration of T-cells [268].

Involvement of MMPs in demyelination and axonal injury has been reported by several groups. Nevertheless, the mechanism of MMP action in MS remains unknown. Newman et al. showed that microinjection of activated MMPs into white matter leads to axonal injury. Of the several MMPs tested, the most potent was MMP-9, followed by MMP-2, and finally MMP-7 [269]. The proposed mechanism of MMP action is* via* degradation of the ECM, since MMPs have an established role in the apoptosis of different cell types by the same mechanism [270]. Additionally, MMP-activated axonal degeneration was hardly observed in peripheral nervous system, possibly due to the presence of resilient ECM and higher TIMPs expression [271].

Beneficial roles of MMPs have also been noted in MS [253]. For example, MMP-9 has a distinctive role in oligodendrocyte process growth [272]. It has been speculated that this could be a cause of reduced remyelination and decreased number of mature oligodendrocytes in MMP-9 and MMP-9/-12 null mice [273].

### 4.6. Other Neurodegenerative Diseases

MMPs have been implicated in other neurodegenerative diseases as well. Huntington's disease (HD) is an inherited neurodegenerative disorder that decreases muscle coordination and mental ability. The disease has been linked to a mutation on chromosome 4 in a gene coding for a protein called huntingtin (Htt). While the exact role of Htt is not clear yet, it has been speculated that proteolysis of mutant Htt participates in the pathology [274]. Besides caspases and calpains acting as proteases in HD, it seems that MMPs also have a distinctive role in cleavage of Htt. Miller et al. have shown that knocking down MMP-10, MMP-14, and MMP-23 in cultured striatal cells expressing mutant Htt diminishes toxicity. Additionally, MMP-10 can directly cleave Htt, and the production of toxic Htt fragments is reduced upon silencing of MMP-10 [77]. Analysis of deceased patients with HD revealed an increase in MMP-9 in comparison to controls, as well as upregulation of cytokine levels (IL-6, IL-8) in cortex and cerebellum [78]. In striatum, the main area affected in HD, only CCL2 and IL-10 were upregulated. Other evidence for involvement of MMP-9 in HD comes from the 3-nitropropionic acid animal model of the disease [79]. The authors showed that MMP-9 is accountable for the BBB disruption that occurs in the disease. Moreover, significantly elevated levels of MMP-9 were found in plasma of patients suffering from HD, as well as in the R6/2 mouse HD model [275]. The authors proposed MMP-9 (along with IL-6, VEGF, and TGF-*β*) as a potential biomarker of HD. As far as TIMPs are concerned, Lorenzl et al. found increased TIMP-1 and TIMP-2 levels in CSF of patients suffering from HD [276].

As far as other neurodegenerative diseases are concerned, different MMPs were found to be dysregulated in people suffering from dementias. Decreased levels of TIMP-2 were found in serum of patients with frontotemporal dementia, and downregulated TIMP-1 was shown in patients with vascular dementia [54]. Intriguingly, patients with vascular dementia have been noted to have higher levels of MMP-9 in CSF even compared to patients suffering from AD [277]. In CSF from patients suffering from Creuztfeldt-Jakob disease, a rare type of dementia, there were increased levels of pro-MMP-9 and active MMP-2, as well as TIMP-1 and TIMP-2 [278].

## 5. Therapeutic Opportunities

**Figure 4:**
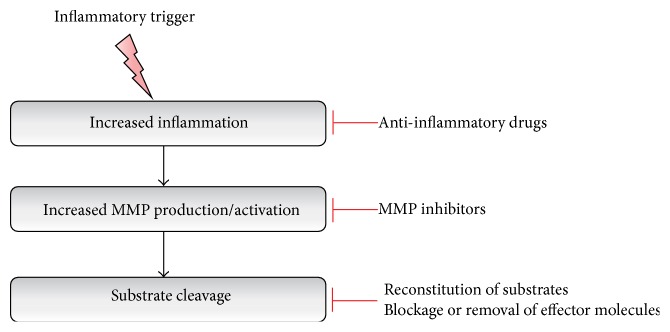
Strategies for targeting MMPs. Inflammatory triggers or protein aggregates in neurodegenerative diseases initiate an inflammatory cascade. At this early stage, various pharmacological anti-inflammatory drugs are effective in eliminating the downstream consequences. Increased inflammation induces and/or activates MMPs, and various broad spectrum inhibitors are available to inhibit MMPs. However, due to the fact that MMPs have both beneficial and detrimental effects, specific MMP inhibition might be a better approach. Finally, it is also possible to interfere at the level of the cleaved substrates, either by reconstitution of crucial substrates or by blockage or removal of effector molecules.

For decades, conventional wisdom has taught us that MMPs play a pivotal role in the dissemination of cancer: they degrade the connective tissue between the cells and allow the cancer cells to leak from the primary site of tumor formation. During the past decade, the role of MMPs became well appreciated in neurodegenerative diseases as well. More than a dozen of MMPs have been shown to be involved in progression of neurodegenerative disorders, thereby opening up the possibility of therapeutically targeting MMPs. In most neurodegenerative disorders, neuroinflammation is observed either before or during the development of the pathological characteristics of the disease. During neuroinflammation, MMPs often increase the permeability of the CNS barriers by destroying the stability of the tight junction proteins or degrading the ECM, which in turn leads to infiltration of immune cells into the brain and cell death [279]. On the other hand, in certain neurodegenerative diseases such as AD and MMPs, and, in particular, MMP-3 and MMP-9, were shown to degrade A*β* plaques [50, 58], justifying the view of MMPs as a double-edged sword. Therefore, drugs that inhibit MMPs could have unforeseen effects that need to be well understood and avoided before we employ them for therapy (Figure 4). MMP activity in neurodegenerative diseases can be inhibited at various stages of disease progression. MMP expression is usually triggered by an inflammatory stimulus (e.g., infection, burns, or protein aggregates), which induces an inflammatory cascade. At this stage, anti-inflammatory drugs will be effective in eliminating the expression or activation of MMPs. Subsequently, the available synthetic broad-spectrum inhibitors might be used to inhibit MMPs (Table 3). However, more-specific MMP inhibitors could be more desirable. Indeed, selective MMP inhibition might avoid the unwanted side effects of broad-spectrum MMP inhibition. MMP inhibitors can be broadly classified as macromolecular inhibitors (including TIMPs and monoclonal antibodies) and both synthetic and natural small molecules [280, 281]. In general, MMP inhibitors act by binding to the Zn^2+^ atom in the active site. Early studies conducted using broad spectrum MMP inhibitors such as batimastat on mice injected with human cancer cells gave compelling results of extending the life of the mice from six- to sevenfold, thereby paving the way for the potential use of MMP inhibitors in other diseases [1]. MMP inhibitors yielded beneficial results in animal studies of lung inflammatory diseases [282], multiple sclerosis [265], meningitis [283–285], vascular dementia [52], stroke [286], acute cerebral ischemia [287, 288], and sepsis [1, 15, 289, 290]. Finally, interfering with the substrates downstream of MMPs might also have therapeutic value.

*Alzheimer's Disease*. The potential use of MMP inhibitors in AD is very speculative and is based on the seemingly beneficial effect of MMP-9, due to its role in degradation of amyloid plaques and hence its contribution to the clearance of A*β* from the brain. Furthermore, it has been reported that MMP-2 can cleave A*β* at the *α*-secretase site [291]. In another similar study, it was reported that MMP-2 also can cleave full-length APP [292], indicating that it can produce *α*-APPs at the plasma membrane or degrade A*β* in the ECM, which leads to reduction of A*β* burden in the brain. In contrast to those reports, other authors were unable to show a similar MMP-2 activity, but they found that MMP-2 might possess *β*-secretase like activity, which might shift the balance towards the amyloidogenic pathway. However, whether MMP-2 can cleave APP remains debatable [291, 293]. Other evidence highlighted the role of MMP-2 and MMP-9. Mice deficient in MMP-2 or MMP-9 appeared to have higher levels of A*β* than wild type animals. Likewise, treatment with the broad-spectrum MMP inhibitor GM6001 resulted in an increase in A*β* in transgenic mice overexpressing the Swedish variant of APP [85]. In an* in vitro* study, GM6001 was shown to block the A*β*-induced alterations in ZO-1 expression and BBB permeability. Similarly, GM6001 was also able to prevent the A*β* oligomer-induced degradation of the blood-CSF barrier integrity [32]. Moreover, in another study on a transgenic mouse model of AD, it was reported that inhibition of MMPs with GM6001 reduced the oxidative stress associated with CAA [86]. TIMPs, the endogenous inhibitors of MMPs, were found to be localized near the A*β* plaques and neurofibrillary tangles of AD-affected brain samples. It has been speculated that MMPs and TIMPs contribute to the evolution of these lesions. Similarly, it has been shown that MMPs are produced in excess at lesion sites by the immune cells surrounded by the effected regions, and that TIMPs might be localized in these places as well to control the activity of MMPs. It is evident that deregulation of TIMPs also leads to progression of AD [294]. The importance of MMPs and TIMPs in AD is not established. Thus, to validate MMPs and TIMPs as possible candidates for therapeutics development, it is important to investigate whether they are amyloidogenic or prevent A*β* accumulation. So far, no MMP inhibitor has been developed successfully as drug for AD. This is mainly because of potential harmful side effects of broad spectrum MMP inhibitor activities, which pose a big hurdle [1].

*Parkinson's Disease*. As far as therapeutic opportunities of MMP inhibition in PD are concerned, Lorenzl et al. reported the expression of MMPs such as MMP-1, MMP-2, and MMP-9 and also TIMP-1 and TIMP-2 in substantia nigra of postmortem PD brain samples [63]. Hence, MMP inhibitors might hold promise for management of PD because death of dopaminergic neurons seems to be linked with release of MMPs. Apoptotic dopaminergic neurons release MMP-3, which in turn activates microglia* in vitro*, indicating that MMP-3 could serve as a signaling molecule as well. The activated microglia release proinflammatory cytokines, such as TNF, that lead to neuronal cell death [64]. Treatment of mouse mesencephalic cells with tetrahydrobiopterin (BH4), a selective dopaminergic neuronal toxin, decreased cell survival. However, when cells were exposed to a selective MMP-3 inhibitor, NNGH (N-isobutyl-N-[4-methoxyphenylsulfonyl]-glycylhydroxamic acid), cell survival was extended* via* the decrease of TNF-*α* release from activated microglial cells [51].

*Amyotrophic Lateral Sclerosis*. Similarly, several hypotheses were proposed regarding the role of MMPs in the development of ALS. Furthermore, selective MMP inhibitors might be potential targets for treatment of ALS. Kiaei et al. reported that by crossing G93A SOD1 mice with MMP-9 knockout mice, immunoreactivity was increased and expression of MMP-9 was elevated in spinal cord tissue of G93A SOD1 mice, a model of familial ALS [73]. Reduced MMP-9 activity was shown to prolong survival in the ALS mouse model expressing mutant SOD1, pointing to MMP-9 as a potential therapeutic target [244]. Generally, MMP-9 stimulates neuronal TNF-*α* by cleaving it from its membrane-bound form, and it also contributes to neuronal cell death by activating other proinflammatory cytokines [73]. Abnormally high levels of MMP-9 and possible degradation of the matrix components contribute to ALS progression [236].

*Multiple Sclerosis*. There are several reports on the use of synthetic MMP inhibitors to ameliorate the symptoms of EAE, and protease inhibitors were used to treat EAE as early as 1982 [295]. MMP activity was shown to increase threefold in the CSF in two acute models of EAE [90]. Broad-spectrum MMP inhibitors such as GM6001 [87, 89], RO31-9790 [90], UK221,316 [88], d-penicillamine [91], and BB1101 [92] were shown to be beneficial in EAE. MMP-9 was shown to be elevated at the lesion sites [82] and in the CSF of MS patients [296]. Similarly, association of MMP-9 with the disruption of the BBB was also reported in MRI studies [297]. When GM6001 was administered after the clinical onset of the disease, it inhibited the development of EAE, and it also reversed the clinical symptoms in SJL/J mice. Likewise, there was a reduction in MMP-9 activity in the treated mice [89]. It was also speculated that MMP inhibition results in restoration of damaged BBB, thereby decreasing the inflammation rather than inhibiting demyelination. In a similar study by Hewson et al., using RO31-9790 reduced the clinical signs in the EAE model of MS when given on the day of disease induction or three days after induction. RO31-9790 was less effective in controlling the disease in animals with more severe clinical signs [90]. BB1101, another broad-spectrum inhibitor, reduced disease severity in Lewis rats [92] and reversed acute symptoms in SJL/J mice [90]. BB1101 was also shown to be effective in chronic relapsing EAE in SJL/J mice, in which BB1101 treatment reduced the glial scar and demyelination. Further, B1101 treatment shifted the cytokine profile from a proinflammatory to an anti-inflammatory state [265]. An antirheumatic drug, d-penicillamine, was also shown to partially protect against EAE in SJL/J mice, but in chronic relapsing EAE in Biozzo mice, treatment with d-penicillamine resulted in attenuation of disease progression after disease induction [91]. The therapeutic efficacy of minocycline, a semisynthetic derivative of tetracycline, was tested in MOG35_55 peptide-induced EAE in C57BL/6 mice [93]. It reduced both the activity and expression of MMP-9 in T-cells and reduced disease severity. In the same study, minocycline inhibited MMP-2. Interestingly, an antioxidant molecule, *α*-lipoic acid, was found to be beneficial in suppressing EAE and reducing disease severity after disease induction. This effect was linked with reduced infiltration of T cells into the CNS, which led to speculation that *α*-lipoic acid could be inhibiting MMP-9 [298]. So far, no molecular mechanism has been identified to explain how MMP inhibition ameliorates MS disease symptoms, but it is speculated that broad-spectrum MMP inhibition inhibits transmigration of immune cells into the CNS* via* the BBB. That would result in reduced demyelination and reduced levels of TNF through inhibition of ADAM17 [299].

## 6. Conclusion

There is currently no clinically available therapy to treat or delay neurodegenerative diseases. Therefore, novel strategies are needed to harness the ability of neuroprotective mechanisms to slow down or stop the progression of the disease in order to prolong the healthy lifespan of patients. More basic research is required to fully understand the diverse roles of MMPs in the pathophysiology of neurodegenerative diseases in order to design specific MMP inhibitors and therapeutic strategies for these chronic diseases of the nervous system.

Although the potential causes and etiology of neurodegenerative diseases remain largely elusive, MMPs clearly have a pivotal role in the progression of neurodegenerative diseases such as AD, PD, ALS, HD, and MS, and their functions in these diseases seem to be much more complex than previously thought. Targeting MMPs will be of much interest for the treatment of these disorders. In most clinical cases, the function of MMPs is difficult to predict. Hence, it is necessary to explore the role of the different MMPs in depth to be able to develop more therapeutic options. More than a dozen MMPs were shown to be involved in neurodegenerative diseases, including MMP-2, MMP-3, and MMP-9, and they seem to be important players in most of the diseases mentioned in this review. The mechanisms of action by which they contribute to the aggravation of neurodegenerative diseases are slowly starting to unfold. MMPs participate in a common pathway of pathological changes in the CNS homeostasis, that is, accumulation of proinflammatory molecules or aggregated proteins and peptides, leading to increased permeability of CNS barriers and consequently to cell death. On the other hand, these enzymes have many vital roles in physiological processes. The dual roles of MMPs hinder efforts to use broad-spectrum MMP inhibitors as therapeutics. However, a strong indication that selective MMP inhibitors could have therapeutic opportunities already exists. Consequently, investigation of MMPs and TIMPs as potential biomarkers and therapeutics in neurodegenerative diseases needs to be continued.

## Figures and Tables

**Table 1:** Classification and structure of MMPs.

Family	MMPs	Structure
Gelatinases	MMP-2, MMP-9	Signal peptide, propeptide, catalytic domain with fibronectin type II motif repeats, hinge region, and hemopexin domain

Matrilysins	MMP-7, MMP-26	Signal peptide, propeptide, and a catalytic domain

Archetypal MMPs	*Stromelysins* MMP-3, MMP-10	Signal peptide, propeptide, a catalytic domain, hinge region, and a hemopexin domain
	*Collagenases* MMP-1, MMP-8, MMP-13, MMP-18	
	*Other* MMP-12, MMP-19, MMP-20, MMP-27	

Furin-activatable MMPs	*Secreted* MMP-11, MMP-21, MMP-28	Signal peptide, propeptide, furin recognition motif, a catalytic domain, hinge region, and a hemopexin domain
	*Type-I transmembrane* MMP-14 (MT1-MMP), MMP-15 (MT2-MMP), MMP-16 (MT3-MMP), and MMP-24 (MT5-MMP)	
	*Type-II transmembrane* MMP-23	
	*GPI-anchored* MMP-17 (MT4-MMP), MMP-25 (MT6-MMP)	

GPI, glycosylphosphatidylinositol; MMP, matrix metalloproteinase.

**Table 2:** Overview of the role of different MMPs in neurodegenerative diseases.

Neurodegenerative disease	MMPs involved	Role of MMPs	Model system	Reference
Alzheimer's disease	MMP-1	Increased in AD patients	Patients	[42]
	MMP-2	Decreased MMP-2 activity and low MMP-9 levels after stimulation with A*β* oligomers	*In vitro* (primary astrocytes)	[43]
		Increased MMP-2 and proinflammatory cytokine levels in the brain	*In vivo *(APP/PS1 mice)	[43]
		Induced upon interaction of A*β* and RAGE	*In vitro* (brain endothelial cells)	[44]
		MMP-2 and MT1-MMP expression observed in reactive astrocytes around plaques	*In vitro *and* in vivo *(Tg-SwDI and Tg2576 mice)	[45]
	MMP-3	Increased expression upon stimulation with A*β*_1–40_	*In vitro* (astrocyte and mixed hippocampal cultures)	[46]
		Increased expression in microglia after stimulation with A*β*_1–42_	*In vitro* (microglial cell line Ra2)	[47]
		Involved in synaptic plasticity	*In vivo *(*rats*)	[48]
		Significantly upregulated plasma and levels correlate with CSF	Patients (plasma and CSF)	[49]
		Ability to degrade A*β*	*In vitro *(APP-CHO cells )	[50]
		Increased expression	*In vivo* (icv injection of A*β* oligomers)	[32]
		Increased BCSFB permeability	*In vivo* (icv injection of A*β* oligomers)	[32]
	MMP-9	Strong expression in microglia, astrocytes, and endothelial cells in the brain	*In vitro *(primary cultured dopaminergic neurons)	[51]
		Biomarker to differentiate AD from dementia	Patients (CSF)	[52]
		Cognitive impairment	Patient samples	[53]
		Elevated serum MMP-9 levels	Patient samples	[54]
		Degrades A*β* fibrils *in vitro* and A*β* plaques in *ex vivo* brain slices	*In vitro* and *ex vivo* (APP/PS1 and APPsw mice)	[55]
		Expression detected in neuronal cytoplasm, neurofibrillary tangles, amyloid plaques, and vascular tissue	Patients (postmortem brain tissue)	[56]
		Detected in astrocytes when treated with soluble and fibrillar A*β*_1–40_ and A*β*_1–42_	*In vitro *(primary astrocytes)	[57]
		MMP-9 can cleave A*β*_1–40_	*In vitro *(isolates from patients brains)	[58]
		Involved in synaptic plasticity	*In vivo *(rats)	[48]
		Increased in hippocampus upon intracerebroventricular injection	*In vivo *(mice)	[59]
		Regulator of NMDA receptor	*In vitro* (hippocampal neurons)	[60]
		BBB disruption, activation of CypA/MMP-9 in pericytes	Patients (CSF)	[61]
	MMP-12	Increase in microglia	*In vitro* (microglial cell line Ra2)	[62]
	MMP-13	Increase in microglia	*In vitro* (microglial cell line Ra2)	[62]

Parkinson's disease	MMP-2	Detected in astrocytes and microglia	Patients	[63]
	MMP-3	Activates microglia	*In vitro *(PC12 cells)	[64]
		MMP-3 dependent ERK signal pathway activation in microglia	Patients (postmortem brain tissue)	[65]
		Induces dopaminergic neuron cell death in mesencephalic neuron-glia mixed culture of wild-type	*In vitro* (neuron-glia mixed culture)	[66]
		Induce production of NO in microglia	*In vitro* (primary mesencephalic cultures from NADPH oxidase null or wild-type mice)	[66, 67]
		MMP-3 secretion by neurons	*In vitro* (primary cultured dopaminergic neurons of wild-type and MMP-3 knockout)	[51]
		Proteolysis of *α*-synuclein	*In vitro* (human dopaminergic neuroblastoma (SK-N-BE) cell line)	[68]
	MMP-9	Increased MMP-9 activity in striatum and substantia nigra after MPTP treatment	*In vivo *(1-methyl-4-phenyl-1,2,3,6-tetrahydropyridine (MPTP) mouse model of PD)	[69]
		MMP-9 was primarily localized in neurons	Patients (postmortem brain tissue)	[63]
		Increased MMP-9 expression substantia nigra	*In vivo *(mouse and monkey models of 1-methyl-4-phenyl-1,2,3,6-tetrahydropyridine- (MPTP-) induced PD)	[70]

Amyotrophic lateral sclerosis	MMP-2	To evaluate ALS disease progression	Patients (serum)	[71]
		Increased BBB permeability	Patients	[72]
	MMP-3	Contributes to motor neuronal cell death	*In vivo *(G93A SOD1 mice)	[73]
	MMP-9	Upregulates neuronal TNF and FasL expression and activation	*In vivo *(G93A SOD1 mice)	[73]
		Dysregulated activity with disease progression	*In vivo *(mutant SOD1 transgenic mice)	[74]
		Low levels of MMP-9 in CSF	Patients (CSF)	[75]
		Elevated in skin and CSF	Patients (skin and CSF)	[71]
		MT-MMP-1/MMP-9 as a marker to distinguish ALS patients from healthy individuals	Patients (serum)	[72]
		Genetic risk factor for ALS	Patients (peripheral blood leukocytes)	[76]

Huntington disease	MMP-10	Cleaves huntingtin	*In vitro* (striatal cell culture expressing mutant Htt)	[77]
	MMP-9	Increased MMP-9 expression	Patients (postmortem brain tissue)	[78]
		Increased MMP-9 expression	*In vivo *(3-nitropropionic acid animal model of the disease)	[79]
	MMP-14	Knockdown of MMP-14 reduces toxicity	*In vitro* (striatal cell culture) expressing mutant Htt)	[77]
	MMP-23	Knockdown of MMP-23 reduces toxicity	*In vitro* (striatal cell culture expressing mutant Htt)	[77]

Multiple sclerosis (MS)	MMP-1	Expression in macrophages, and weak expression in astrocytes near necrotic lesions	Patients (active lesion sites of postmortem brain samples)	[80]
		Increased mRNA levels	Patients (monocytes)	[81]
	MMP-2	Expression in macrophages and weak expression in astrocytes near necrotic lesions	Patients (active lesion sites of postmortem brain samples)	[80]
	MMP-3	Expression in endothelial cells	Patients (active lesion sites of postmortem brain samples)	[80]
		Increased mRNA levels	Patients (monocytes)	[81]
	MMP-7	Secreted by activated macrophages	Patients (active lesion sites of postmortem brain samples)	[82]
		Increased mRNA levels	Patients (monocytes)	[81]
	MMP-9	Secreted by blood vessels	Patients (active lesion sites of postmortem brain samples)	[82]
		Increased mRNA levels	Patients (monocytes)	[81]
		Expression in macrophages and weak expression in astrocytes near necrotic lesions	Patients (active lesion sites of postmortem brain samples)	[80]
		Secreted by T-cells and macrophages, contributes to tissue damage surrounding lesion	Patients (CSF samples from both RRMS and PPMS patients)	[83]
		Increased levels of MMP-9 in serum along with TIMP-1 and TIMP-2	Patients (serum)	[84]

A*β*, *β*-amyloid; AD, Alzheimer's disease; ALS, amyotrophic lateral sclerosis; APP, amyloid precursor protein; BBB, blood-brain barrier; BCSFB, blood-CSF barrier; CSF, cerebrospinal fluid; Cyp A, cyclophilin A; EAE, experimental autoimmune encephalomyelitis; ERK, extracellular signal-regulated kinases; FasL, Fas ligand; icv, intracerebroventricular; MMP, matrix metalloproteinase; MPTP, 1-methyl-4-phenyl-1,2,3,6-tetrahydropyridine; NO, nitric oxide; PS1, presenilin-1; NMDA, N-methyl-D-aspartate; PPMS, primary progressive multiple sclerosis; RAGE, receptor for advanced glycation end products; RRMS, relapsing-remitting multiple sclerosis; SOD, superoxide dismutase; TIMP, tissue inhibitor of metalloproteinases; TNF, tumor necrosis factor.

**Table 3:** Overview of *in vitro* and *in vivo* MMP inhibitor studies in Alzheimer's disease, Parkinson's disease and multiple sclerosis.

Neurodegenerative disease	Type of MMP inhibitor	Inhibitor	Phenotype	Model system/Source	References
Alzheimer's disease	Broad spectrum	GM6001	Increased brain interstitial fluid A*β* levels	Transgenic mice overexpressing the Swedish variant of APP	[85]
	Broad spectrum	GM6001	Protection from BBB permeability	*In vitro *(brain endothelial cells)	[33]
	Broad spectrum	GM6001	Protection from BCSFB permeability	*In vivo *(icv injection with A*β* oligomers)	[32]
	Broad spectrum	GM6001	Reduced oxidative stress	Transgenic mouse model of AD	[86]

Parkinson's disease	Selective	NNGH (N-isobutyl-N-[4-methoxyphenylsulfonyl]-glycylhydroxamic acid)	Decrease of TNF release from microglial cells and increased cell survival	*In vitro* (mouse mesencephalic cells)	[51]

Multiple sclerosis	Broad spectrum	GM6001	Reversal of clinical signs of EAE	EAE	[87]
			Reduced *α*4 integrin-mediated transmigration	EAE	[88]
			Reversed clinical symptoms and reduced MMP-9 activity	SJL/J mice	[89]
	Broad spectrum	RO31-9790	Reduced clinical severity of adoptively transferred EAE	EAE	[90]
	Broad spectrum	UK221,316	Reduced *α*4 integrin-mediated transmigration and EAE	EAE	[88]
	Broad spectrum	d-pencillamine	Reduced mortality and morbidity rates	EAE	[91]
	Broad spectrum	BB1101	Reduces clinical signs and weight loss in an acute EAE	Lewis rats	[92]
	Broad spectrum	Minocycline	Reduced activity and the expression of MMP-9 in T-cells	EAE	[93]
			Inhibits MMP-2	EAE	[94]
		*α*-lipoic acid	Reduced infiltration of T-cells and reduced MMP-9 activity	EAE	[95]

A*β*, *β* amyloid; AD, Alzheimer's disease; BBB, blood-brain barrier; BCSFB, blood-CSF barrier; CSF, cerebrospinal fluid; EAE, Experimental autoimmune encephalomyelitis; MMP, matrix metalloproteinase; TNF, tumor necrosis factor.

## Abbreviations

AD: Alzheimer's disease
AGD: Agyrophilic grain disease
AGE: Advanced glycation end products
AICD: APP intracellular domain
ALS: Amyotrophic lateral sclerosis
APC: Antigen presenting cells
APOE: Apolipoprotein E
APP: Amyloid precursor protein
A*β*: *β*-amyloid
BBB: Blood-brain barrier
BCSFB: Blood-cerebrospinal fluid barrier
BH4: Tetrahydrobiopterin
BSCB: Blood-spinal cord barrier
CAA: Cerebral amyloid angiopathy
CaN: Calcineurin
CBD: Corticobasal degeneration
CNS: Central nervous system
CRP: C-reactive protein
CTFs: C-terminal fragments
CypA: Cyclophilin A
DLB: Dementia with Lewy bodies
EAE: Experimental autoimmune encephalomyelitis
ECM: Extracellular matrix
FTDP: Frontotemporal dementia with parkinsonism-17
FTLD-TDP: Frontotemporal lobar degeneration with TDP proteinopathy
HD: Huntington's disease
Htt: Huntingtin
icv: Intracerebroventricular
IL: Interleukin
MHC: Major histocompatibility complex
MMP: Matrix metalloproteinase
MPTP: 1-Methyl-4-phenyl-1,2,3,6-tetrahydropyridine
MRI: Magnetic resonance imaging
MS: Multiple sclerosis
MSA: Multiple system atrophy
NFT: Neurofibrilary tangles
NO: Nitric oxide
PD: Parkinson's disease
PiD: Pick's disease
PSEN: Presenilin
PSP: Progressive supranuclear palsy
RAGE: Receptor for advanced glycation end products
ROS: Reactive oxygen species
TACE: Tumor necrosis factor-*α*-converting enzyme
TBI: Traumatic brain injury
TDP-43: Transactive response DNA-binding protein 43
TGF-*β*: Transforming growth factor-*β*
TIMP: Tissue inhibitor of metalloproteinases
TJ: Tight junctions
TNF*α*: Tumor necrosis factor *α*
ZO1: Zonula occludens-1.
